# 964. Impact of the COVID-19 Pandemic on Bedside Medical Education: A Mixed-Methods Study

**DOI:** 10.1093/ofid/ofab466.1159

**Published:** 2021-12-04

**Authors:** Eva Clark, Jennifer Freytag, Sylvia J Hysong, Bich Dang, Thomas P Giordano, Prathit A Kulkarni

**Affiliations:** 1 Baylor College of Medicine, Houston, Texas; 2 Center for Innovations in Quality, Effectiveness, and Safety, Houston, Texas; 3 Baylor College of Medicine / Michael E. DeBakey VA Medical Center, Houston, TX

## Abstract

**Background:**

The COVID-19 pandemic obligated academic medical programs to substantially alter the traditional Internal Medicine (IM) rounding model to decrease risk of inpatient nosocomial viral transmission. Our study aimed to describe how IM rounding practices changed during the COVID-19 pandemic and to understand the impacts of these changes on medical education.

**Methods:**

We conducted a two-phase, mixed-methods study of inpatient IM rounding team practices at a large academic hospital in Houston, TX. In the first phase (January-February 2021), we organized and audio-recorded 4 virtual (Zoom) focus groups. Each included 5-6 rounding team members, divided by: attendings; senior residents; interns; and medical and physician assistant students. In the second phase (March-May 2021), we performed 6 direct observations of IM teams during rounds. Two observers systematically recorded variables such as time spent on non-bedside versus bedside rounds, number of each team member type entering patient rooms for bedside teaching, and types of personal protective equipment (PPE) worn.

**Results:**

Topics discussed during focus groups included comparisons of rounding team size, rounding duration, physical distancing and PPE use, bedside education, communication methods, and patient safety before and after March 2020. Perceptions of changes in each topic were generally consistent across groups (Table 1). Direct observation data showed that team rounding styles remained diverse in the proportion of rounding time spent in an office versus on the wards, and in the number and types of team members entering patient rooms. IM team members uniformly wore respiratory PPE when entering all patient rooms; use of eye protection varied. Teams spent more total time discussing patients with or suspected to have COVID-19 compared to patients without COVID-19 (median 24 min versus 13 min, p< 0.0001).

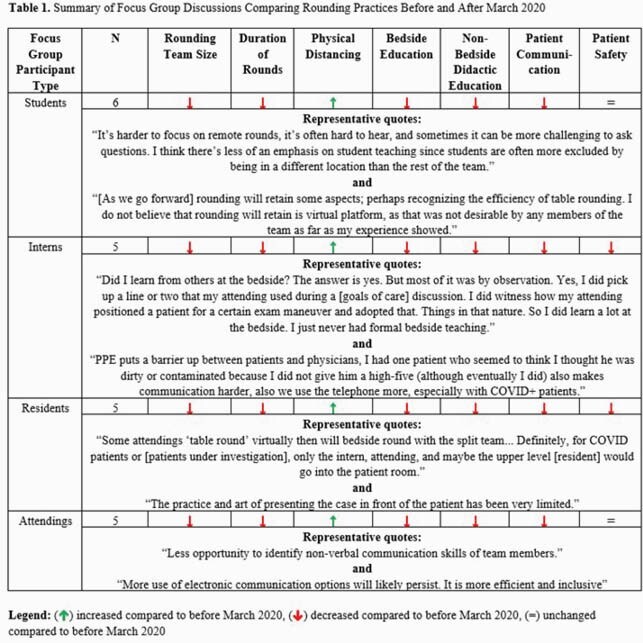

**Conclusion:**

Our results suggest that the COVID-19 pandemic adversely impacted bedside medical education, even into Spring of 2021. Conclusions from this study can be used to 1) address educational gaps related to COVID-19 pandemic-associated rounding changes and 2) create innovative methods of providing high-quality clinical education that will be minimally impacted by future respiratory virus pandemics.

**Disclosures:**

**Prathit A. Kulkarni, M.D.**, **Vessel Health, Inc.** (Grant/Research Support)

